# Intracerebroventricular injection of leukotriene B_4 _attenuates antigen-induced asthmatic response via BLT1 receptor stimulating HPA-axis in sensitized rats

**DOI:** 10.1186/1465-9921-11-39

**Published:** 2010-04-20

**Authors:** Shui-Juan Zhang, Yang-Mei Deng, Yi-Liang Zhu, Xin-Wei Dong, Jun-Xia Jiang, Qiang-Min Xie

**Affiliations:** 1 Zhejiang Respiratory Drugs Research Laboratory of State Food and Drug Administration of China, Medical Science College of Zhejiang University, Hangzhou, China; 2 Department of Pharmacology, Zhejiang Traditional Chinese Medicine University, Hangzhou, China

## Abstract

**Background:**

Basic and clinical studies suggest that hypothalamic-pituitary-adrenal (HPA) axis is the neuroendocrine-immnue pathway that functionally regulates the chronic inflammatory disease including asthma. Our previous studies showed corresponding changes of cytokines and leukotriene B_4 _(LTB_4_) between brain and lung tissues in antigen-challenged asthmatic rats. Here, we investigated how the increased LTB_4 _level in brain interacts with HPA axis in regulating antigen-induced asthmatic response in sensitized rats.

**Methods:**

Ovalbumin-sensitized rats were challenged by inhalation of antigen. Rats received vehicle, LTB_4 _or U75302 (a selective LTB_4 _BLT1 receptor inhibitor) was given via intracerebroventricular injection (i.c.v) 30 min before challenge. Lung resistance (*R*_L_) and dynamic lung compliance (*C*_dyn_) were measured before and after antigen challenge. Inflammatory response in lung tissue was assessed 24 h after challenge. Expression of CRH mRNA and protein in hypothalamus were evaluated by RT-PCR and Western Blot, and plasma levels of adrenocorticotropic hormone (ACTH) and corticosterone (CORT) were measured using the ELISA kits.

**Results:**

Antigen challenge decreased pulmonary function and induced airway inflammation, evoked HPA axis response in sensitized rats. Administration of LTB_4 _via i.c.v markedly attenuated airway contraction and inflammation. Meanwhile, LTB_4 _via i.c.v markedly increased CORT and ACTH level in plasma before antigen challenge, and followed by further increases in CORT and ACTH levels in plasma after antigen challenge in sensitized rats. Expression of CRH mRNA and protein in hypothalamus were also significantly increased by LTB_4 _via i.c.v in sensitized rats after antigen challenge. These effect were completely blocked by pre-treatment with BLT1 receptor antagonist U75302 (10 ng), but not by BLT2 antagonist LY255283.

**Conclusions:**

LTB_4 _administered via i.c.v down-regulates the airway contraction response and inflammation through activation of the HPA axis via its BLT1 receptor. This study expands our concept of the regulatory role of intracranial inflammatory mediators in inflammatory diseases including asthma. The favourable effects of LTB_4 _on the HPA axis may help to explain the phenomenon of self-relief after an asthmatic attack.

## Background

Central nervous system (CNS) and neuroendocrine-immune systems (NEI) are the two major systems which respond adaptively to the numerous challenges to maintain the physiological homeostasis. The adaptive responses could be impaired by some physical and psychological stressors in neuroendocrine-immune feedback system. Such dysfunction could also contribute to the pathogenesis of allergic or autoimmune diseases [1]. The studies on the cross-talk between neuroendocrine and immune systems added further evidences that interactions among the neural, neuroendocrine and immune systems are bidirectional [1-3]. Recent studies have shown that this bidirectional cross-talk is based on the secretion of pro-inflammatory factors including mediators and cytokines, hormones, neurotransmitters and neuropeptides [4-6]. The hypothalamic-pituitary-adrenal (HPA) axis is the major pathway in NEI, hypothalamus secretes corticotropin releasing hormone (CRH) when the HPA axis is activated. This molecule travels to the anterior pituitary gland, which responds to its presence by secreting a pulse of adrenocorticotropin hormone (ACTH). The ACTH signal is carried through the peripheral circulation to the adrenal glands, which synthesize and release cortisol and lead to reduction of inflammation.

Leukotriene (LT) B_4 _is a product of the action of LTA_4 _hydrolase (LTA_4_-H) on LTA_4 _in 5-lipoxygenase (5-LO) pathway. It is a potent leukocyte chemoattractant and activator, which plays an important role in modulating immune and inflammatory responses [7]. An early study showed that LTB_4 _increases CRH secretion in explanted and cultured hypothalamus via autocrine/paracrine or as endocrine factor [8]. Further studies found that inflammatory mediator such as IL-1, IL-6 can activate the HPA axis and regulate the inflammatory response in periphery [9]. From previous work, we found that the changes of Th1/Th2 paradigm (ratio of interferon [IFN]-gamma/interleukin [IL]-4 decreased) [10], and the content of LTB_4 _in the cerebral cortex increases corresponding to their changes in bronchoalveolar lavage fluid (BALF) or lung tissue in inflammatory status of asthmatic rats [11]. Also, the expression of 5-LO and LTA_4_-H mRNA in cerebral cortex of asthmatic rats are significantly higher than those of control rats [12]. All these findings indicate that the changes of these proinflammatory mediators in the CNS may have pathophysiologic effects in asthmatic rats.

So far, it is unclear how LTB_4 _in the CNS regulates inflammation in lung tissue of asthma. Based on these studies, we postulate that the increase of LTB_4 _in brain activates NEI, which may regulate asthmatic response in rats. To explore this hypothesis, rats were actively sensitized with ovalbumin (OVA), and LTB_4 _was administered via intracerebroventricular injection (i.c.v). The pulmonary function and inflammatory cell infiltration in lung were evaluated. Meanwhile, the HPA axis activity was also explored by measuring CRH mRNA and protein expression in hypothalamus, corticosterone (CORT) and ACTH level in plasma during antigen challenge in sensitized rats.

## Methods

### Animal and study design

Sprague-Dawley (SD) rats of either sex weighing 180~200 g were purchased from Laboratory Animal Center in Medical Science College of Zhejiang University (Grade II, Certificate No. 220010014). All animals were housed in Plexiglas cages and kept on a 12/12 h light-dark cycle in temperature and humidity controlled rooms, standard laboratory food and water were provided ad libitum. Food was withheld 8 hours before the experiments, with free access to water. Unless otherwise indicated, Animal treatments were strictly in accordance with the China Community Guidelines for the Use of Experimental Animals and the National Institutes of Health Guide for the Care and Use of Laboratory Animals. Rats were assigned to different treatments, Vehicle-sham group was as normal control, ovalbumin (OVA) sensitized and challenged rats were treated as asthmatic model (Vehicle-OVA), the effects of LTB_4 _(LTB_4_-OVA) via i.c.v on asthmatic rats were evaluated. And U75302, the selective BLT1 receptor antagonist, was administered via i.c.v alone (U75302-OVA) or 5 min before LTB_4 _dosing (U75302-LTB4-OVA).

### Sensitizing procedures

To sensitize the rats, 1 mg OVA (Grade V, Sigma Chemical Co., St. Louis, MO) absorbed in 100 mg aluminium hydroxide adjuvant in 1.0 ml saline. Each rat was intraperitonealy injected (i.p) with 0.5 ml, and subcutaneously injected (s.c) with 0.05 ml/site at four footpads (4 sites), neck (one site), back (3 sites), and two groins (2 sites), respectively. Then, every rat was injected (i.p) with 1 × 10^10 ^bordetella pertussis adjuvant (Shanghai Institute of Biological Products, China) on experiment day 0. Normal control (vehicle-sham) rats were injected with only aluminium hydroxide adjuvant in saline following the same protocol. At day 14 after sensitization, the rats were placed in a 45 cm × 45 cm × 15 cm plastic box and challenged by exposure to an aerosol of OVA (20 mg/ml in saline), which was generated in a jet nebulizer (particle size 1-5 μm; BARI, MASTER, Germany) for 20 min, and repeated daily to day 21 after sensitization. Vehicle-sham rats were similarly exposed to an aerosol of saline.

### Intracerebroventricular injection

After 10% chloral hydrate (3 ml/kg i.p) anaesthesia, the animal's head was fixed in a stereotaxic apparatus (SR-6N, Narishige, Japan). The procedure of i.c.v. injection was in accordance with rat brain graph described by George Paxious and Charles Watson, and with minor improvement as described by Mauser et al [13]. In brief, a midline incision was made from a point just posterior to the eyes to about 3 cm caudal, and the overlying connective tissue was removed to expose the skull. A small hole (about 2 mm in diameter) was opened perpendicularly to the skull, -1.0 or -1.5 mm anterior and 1.5 mm lateral to the bregma by using a dental drill (Minimo, Japan). A stainless steel guide cannula (internal diameter, 0.5 mm; length, 1.5 cm) was then slowly and vertically lowered to a depth of 3.8 mm from the dura into lateral ventricles. The guide cannula was then held in place by dental cement (oral cavity drugs and materials of Wuhan University, China) with a small anchor screw. The scalp was sutured and the animals were left to recover for 1 week before the first antigen challenge. All injections through the i.c.v. were made with a Hamilton syringe (Reno, NV, U.S.A.) and in a 10 μl volume of artificial CSF. In vehicle-sham or LTB_4_-OVA group, vehicle 10 μl or LTB_4 _10 ng (Sigma Chemical Co., St. Louis, MO) (Cayman Chemical) was administered via i.c.v 30 min before the antigen challenge from day 14 to day 21. In the U75302-LTB_4_-OVA group, an extra dose of U75302 10 ng was administered via i.c.v 5 min before LTB_4 _dosing.

### Measurement of pulmonary function

The lung function was assessed 30 min before and after LTB_4 _i.c.v on day 21 after sensitization as described previously [14]. Briefly, each anaesthetized rat was placed supine inside a Plexiglas whole-body plethysmograph. The flow rate was monitored with a Fleisch tube connected to the airway in a pressure transducer. Changes in lung volume were measured by detecting pressure changes in the plethysmographic chamber through a port in the connecting tube with a pressure transducer. To measure pleural pressure, a needle (No. 16) with a multiholed tip was directly inserted into the pleural cavity (between fourth and fifthly rib at left thorax) through a port in the connecting tube with a differential pressure transducer. Transpulmonary pressure was calculated as the difference between mouth and pleural pressure. All signals of pressure transducers were continuously computed (MedLab, Nanjing Biotech Instruments, China) by fitting flow, volume, and pressure to an equation of motion. For antigen challenge, OVA 20 mg/ml dissolved in saline aerosolized by a jet nebulizer (BARI Co. Ltd, Germany) for 5 min. The respiratory waveform was monitored for 15 min and maximal changes from baseline for each parameter were recorded by the MedLab. The results were shown as airway resistance (*R*_*L*_) and dynamic lung compliance (*C*_*dyn*_) value before and after antigen challenge.

### Preparation of bronchoalveolar lavage fluids

Twenty-four hours after the final OVA challenge, rats were anesthetized with urethane (2 g/kg, i.p.). Bronchoalveolar lavage fluids (BALF) were obtained via tracheal tube by washing of the right lung with 1 ml of sterilized saline containing 1% bovine serum albumin (BSA) and 5 IU/ml heparin for three times. The total number of cells in the BALF was counted. The BALF was centrifuged for 10 min at 400 × g at 4°C. Two hundred cells from the cell suspension were stained by Wright-Giemsa and classified using a light microscope. The results were expressed as the numbers of each type of cell population in 1 ml of BALF.

### Lung histopathology

The left lung was fixed in 10% neutral formalin for 7 days. Sections of 5 μm thickness were prepared and stained with hematoxylin-eosin (H&E). To determine the severity of inflammatory cell infiltration, peribronchial eosinophil cell number was blindly counted and the severity was evaluated by a 5 point scoring system described previously [15]. Briefly, the scoring system was 5-marked, 4-moderate, 3-medium, 2-mild, 1-minimal and 0-no eosinophil cells.

### ACTH and CORT assay

Blood samples were collected in heparin-coated tubes via the tail vein 30 min before, 30 min after LTB_4 _i.c.v, and 30 min after final antigen challenge. Blood samples were centrifuged at 2000 × g to separate plasma at 4°C for 15 min. All samples were stored at -80°C until analysis. The levels of ACTH and CORT in plasma were measured using a commercial ELISA kit (USCN-LIFE™, China) by following the manufacturer's instructions.

### Isolation of hypothalamus

Rats were euthanized and then decapitated 24 h after the final antigen challenge. Hypothalamus was dissected with the following limits: anterior border of the optic chiasm, anterior border of the mamillary bodies, and lateral hypothalamic sulci. The depth of dissection was approximately 3 mm. The hypothalamus was then quickly frozen in liquid nitrogen and preserved at -80°C for extraction of RNA or protein.

### Reverse transcription and polymerase chain reaction

Total RNA was isolated with TRIzol Reagent (Invitrogen, USA). First-strand cDNA was generated from 4 μg of total RNA using oligo-dT to prime the reverse transcription according to the supplied protocol (Invitrogen). PCR were performed using commercial PCR reagents Kit (TaKaRa) in a gradient thermal cycler PCR machine (Eppendorf, Germany). Rat CRH was amplified using primers 5'-TCACCTTCCACCTTCTGAGG-3' and 5'-GGAAATGAAATGTTGCGCTT-3' (317 bp, intron spanned) for 35 cycles (94°C 30 s, 58°C 45 s, 72°C 45 s). Primers for rat GAPDH were 5'-ACCACCATGGAGAAGGCTGG-3' and 5'-CACAGTGTAGCCCAGGATGC-3' (528 bp, intron spanned) for 28 cycles (94°C 30 s, 58°C 30 s, 72°C 60 s). Aliquots of polymerase chain reaction products were separated by electrophoresis on 1.5% agarose gels and visualized with ethidium bromide, and the PCR product bands were quantified by using UVP Gel Documentation system (UVP, Upland, CA). Results were expressed as a ratio of densitometry value of CRH to that of GAPDH.

### Western Blot

CRH protein expression in hypothalamus was assessed as described by Meloni EG et al [16]. 20 μg protein/lane (determined by protein assay; Eppendorf Biophotometer, Germany) was loaded onto a 6-well, 12% Bis-Tris gel (Invitrogen) and electrophoresed in 1 × 2-N-morpholino-ethane sulfonic acid (MES)-SDS running buffer (Invitrogen). After electrophoretic transfer, the polyvinylidene fluoride (PVDF) membranes were cut to isolate the beta-actin band as well as the lower molecular weight CRH band. Membranes were incubated in blocking buffer (5% non-fat dry milk in PBS and 0.1% Tween 20; PBS-T) overnight at 4°C and then incubated with either polyclonal rabbit anti-CRH (1:200; Santa Cruz Biotechnology) or rabbit anti-beta-actin (1:1,000; Cell Signaling Technology) antibody diluted in PBS-T for 2 h at room temperature. After incubation in secondary antibody (HRP-conjugated goat anti-rabbit IgG, 1:5000; Li-cor Biosciences) diluted in blocking buffer for 2 h, immunoreactivity was visualized with chemiluminescence using an Image Station (Odyssey, American Gene Co.). Specific CRH band was determined by the synthetic rat CRH (rCRH, American peptide Company). Immunoblots were quantified by image analysis (UVP, Upland, CA) and data were expressed as a ratio of densitometry value of CRH to that of beta-actin.

### Statistical analysis

Numerical data were presented as means ± S.D. Statistical calculations were performed using SigmaStat software (SigmaStat 2.0, SPSS Inc., Chicago, IL, USA), ANOVA and Student-Newman-Keuls multiple comparisons test were used to calculate the significance of differences of respiratory function, inflammatory cells in BALF, levels of CORT and ACTH in plasma, and expression of CRH in hypothalamus. A non-parametric test, the Mann-Whitney U-test, was used to compare differences in eosinophil infiltration in airway. Significance was assessed at the *P *< 0.05 level.

## Results

### The changes of airway resistance and dynamic lung compliance

**Figure 1:**
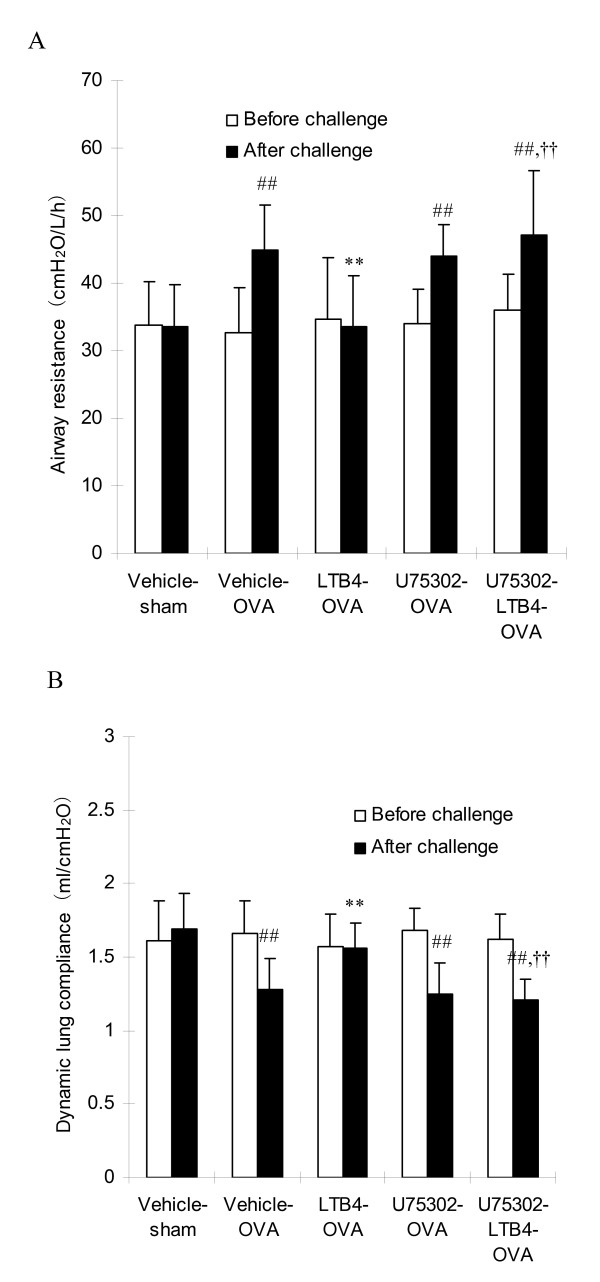
**LTB_4 _via i.c.v attenuates the antigen-induced changes of pulmonary function in rats, and U75302 blocks the inhibitory effect of LTB_4_**. On day 21 after ovalbumin sensitization, rats were challenged for 5 min with aerosolized 2% ovalbumin and the airway resistance (*R*_*L*_) (A) and dynamic lung compliance (*C*_*dyn*_) (B) were measured before and 5 min after the antigen challenge. Data are expressed as the mean ± S.D of vehicle-sham (n = 8), vehicle-OVA (n = 10), LTB_4_-OVA (n = 9), U75302-OVA (n = 9) and U75302-LTB_4_-OVA (n = 10). ^##^*P *< 0.01 vs the vehicle-sham group; **P < 0.01 vs the vehicle-OVA group; ^††^*P *< 0.01 vs the LTB_4_-OVA group.

Compared with the baseline values, saline inhalation did not show effects on the airway resistance (*R*_L_) and dynamic lung compliance (*C*_dyn_) in both vehicle-sham rats and OVA-sensitized rats (data not shown). Bronchial challenge of OVA induced significant increase of *R*_L _and decrease of *C*_dyn _with maximal response at 4~5 min in the vehicle-OVA group. These responses were suppressed by treatment of LTB4 via i.c.v as shown in the LTB4-OVA group (P < 0.01), but not by U75302. However, U75302 at 10 ng via i.c.v. completely blocked the inhibitory effects of LTB4 on antigen-induced increase of *R*_L _and decrease of *C*_dyn _in LTB4-U75302-OVA group (Fig. 1).

### Airway inflammation

**Figure 2:**
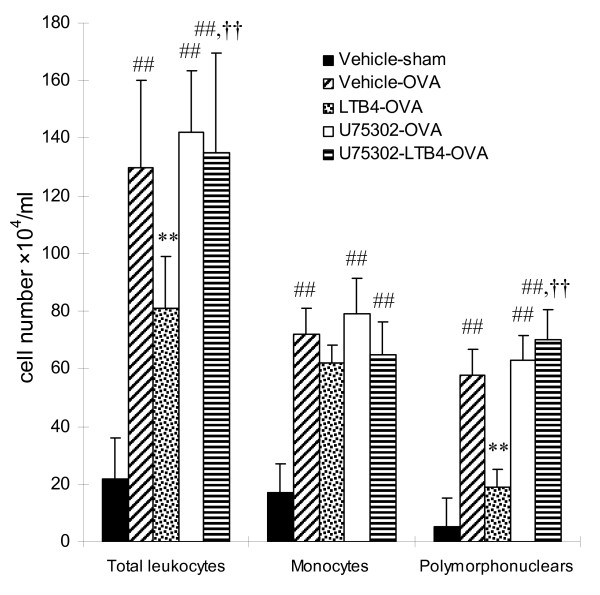
**LTB_4 _via i.c.v attenuates the antigen-induced increases of inflammatory cell in the BALF, and U75302 blocks the inhibitory effect of LTB_4_**. Total inflammatory cells in BALF were counted, and cell classification was performed on a minimum of 200 cells to classify monocytes (lymphocytes and macrophages) and polymorphonuclear cells (eosinophils and neutrophils) 24 hr after the final antigen challenge. Data are expressed as the mean ± S.D. of vehicle-sham (n = 8), vehicle-OVA (n = 10), LTB_4_-OVA (n = 9), U75302-OVA (n = 9) and U75302-LTB_4_-OVA (n = 10). ^##^*P *< 0.01 vs the vehicle-sham group;**P < 0.01 vs the vehicle-OVA group; ^††^*P *< 0.01 vs the LTB_4_-OVA group.

To further study the effect of LTB_4 _i.c.v on antigen-induced airway inflammation, we evaluated the inflammatory cell infiltration in OVA sensitized rats. 24 h after the final OVA challenge, inflammatory cells including polymorphonuclear (PMN) cells (eosinophils and neutrophils) and monocytes (lymphocytes and macrophages) in BALF were counted. The total inflammatory cells in BALF of vehicle-OVA rats was 6-fold greater than that in vehicle-sham rats. LTB4 10 ng (i.c.v) significantly decreased the total inflammatory cells in BALF. Classification of these inflammatory cells showed that in vehicle-OVA rats, numbers of PMN and monocytes in the BALF increased 11.6 and 4.2 fold, respectively, as compared with those observed in vehicle-sham rats (Fig. 2). LTB_4 _10 ng (i.c.v) significantly decreased PMN in BALF. U75302 10 ng itself (i.c.v) did not alter the infiltration of inflammatory cells in airways, but almost fully blocked inhibitory effects of LTB4 on the inflammatory cell numbers in BALF (*P *< 0.01).

### Eosinophil infiltration in lung tissues

**Figure 3:**
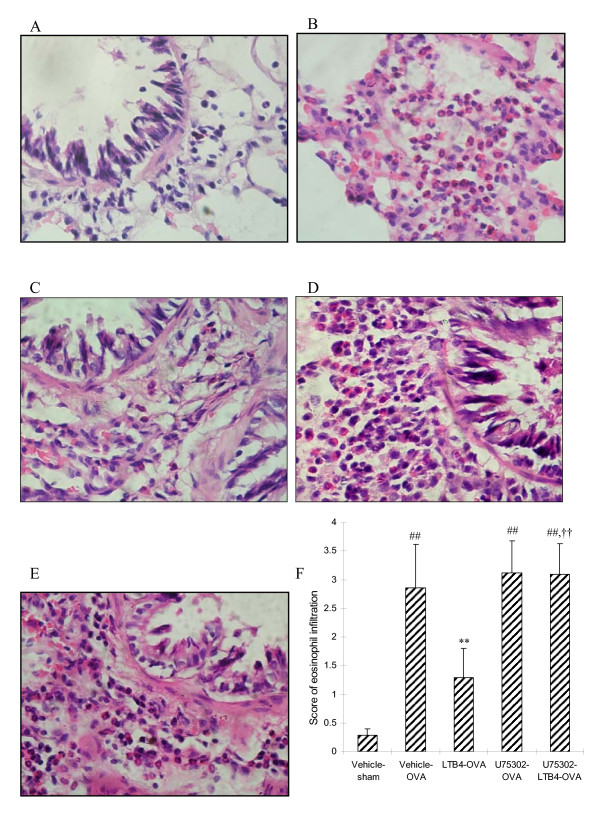
**Lung histopathology**. Haematoxalin and eosin-stained lung tissues were evaluated and scored for eosinophil cell infiltration. Compared with vehicle-sham group (A), marked infiltration of eosinophil cells in peribronchiolar space and perivascular space were observed in the vehicle-OVA (B), there were no significant eosinophil cell infiltration presented in the group of LTB_4_-OVA (C). U75302-OVA (D) and U75302-LTB_4_-OVA (E) did not show any attenuation of eosinophil infiltration. Scores of eosinophil cell infiltration were graded on the basis of severity of inflammation (F). ^##^*P *< 0.01 vs the vehicle-sham group;**P < 0.01 vs the vehicle-OVA group;^††^*P *< 0.01 vs the LTB_4_-OVA group.

Effect of LTB_4 _via i.c.v on antigen-induced inflammatory response was also evaluated by lung histopathology. Lung tissues were harvested 24 h after the final OVA challenge. The vehicle-OVA rats exhibited an obvious eosinophil cell infiltration into the peribronchiolar and perivascular connective tissues as compared with vehicle-sham rats. LTB_4 _10 ng (i.c.v) markedly inhibited the OVA-induced eosinophil infiltration as compared with vehicle-OVA rats (*P *< 0.01), and the inhibitory effect of LTB_4 _was blocked by U75302 (Fig. 3).

### Plasma CORT and ACTH concentrations

**Figure 4:**
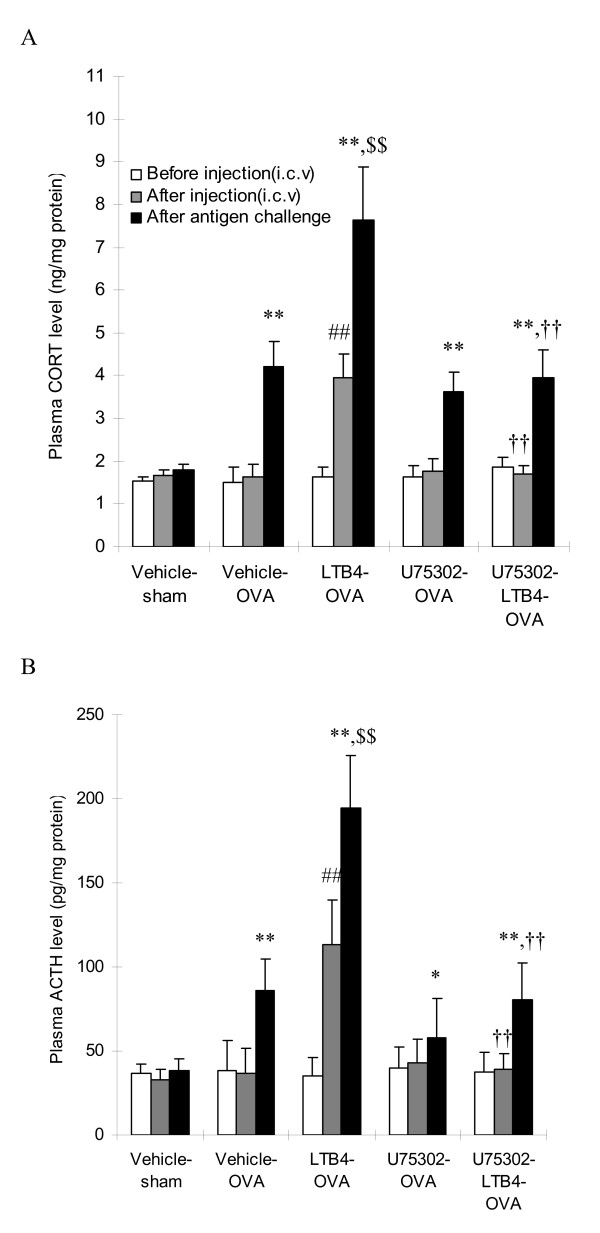
**LTB_4 _via i.c.v injection increases CORT and ACTH levels in rat plasma**. CORT (A) and ACTH (B) levels in plasma were measured using commercial ELISA kits at 30 min before and after LTB_4 _i.c.v, and 30 min after antigen challenge. Data are expressed as the mean ± S.D (n = 6 in each group). ^##^*P *< 0.01 vs the basal level (pretreatment); **P < 0.01 vs after antigen challenge;^$$^*P *< 0.01 vs before antigen challenge; ^††^*P *< 0.01 vs the LTB_4_-OVA group.

To further test the hypothesis that LTB_4 _exert its inhibitory effect via activation of HPA axis, we measured the level of CORT and ACTH in plasma 30 min before and after LTB_4 _administration, and 30 min after antigen challenge. Plasma CORT and ACTH concentrations did not differ significantly before LTB_4 _i.c.v treatment in all groups (Fig. 4). LTB_4 _via the i.c.v markedly increased plasma CORT and ACTH secretion rate in the LTB_4_-OVA group to 2.4 and 3.2 folds of the basal rate, respectively. However, U75302, at the dose of 10 ng (i.c.v), markedly blocked LTB_4 _induced increase of secretion of CORT and ACTH in plasma (*P *< 0.01). Interestingly, plasma CORT and ACTH concentrations in all treatment groups increased significantly 30 min after antigen challenge, and LTB_4 _at 10 ng (i.c.v) additionally increased the concentration of plasma CORT by 92.6%(P < 0.01) and ACTH by 71.5% (*P *< 0.01) after antigen challenge as compared with that after LTB4 i.c.v treatment. Furthermore, compared with LTB_4_-OVA group, pretreatment with U75302 at 10 ng also suppressed LTB_4 _i.c.v induced increase of CORT and ACTH after antigen challenge. On contrast, LY255283, a BLT2 antagonist (Cayman Chemical), did not significantly block LTB_4 _effects even at a large dose (50 ng/kg, i.c.v) (data not shown). Compared with the vehicle-OVA group, U75302 alone at 10 ng (i.c.v) only decreased CORT (*P *> 0.05) and ACTH(*P *> 0.05) level for around 15% to 20% in plasma after antigen challenge in U75302-OVA group when comparing with vehicle-OVA group.

### Expression of CRH mRNA and protein in hypothalamus

**Figure 5:**
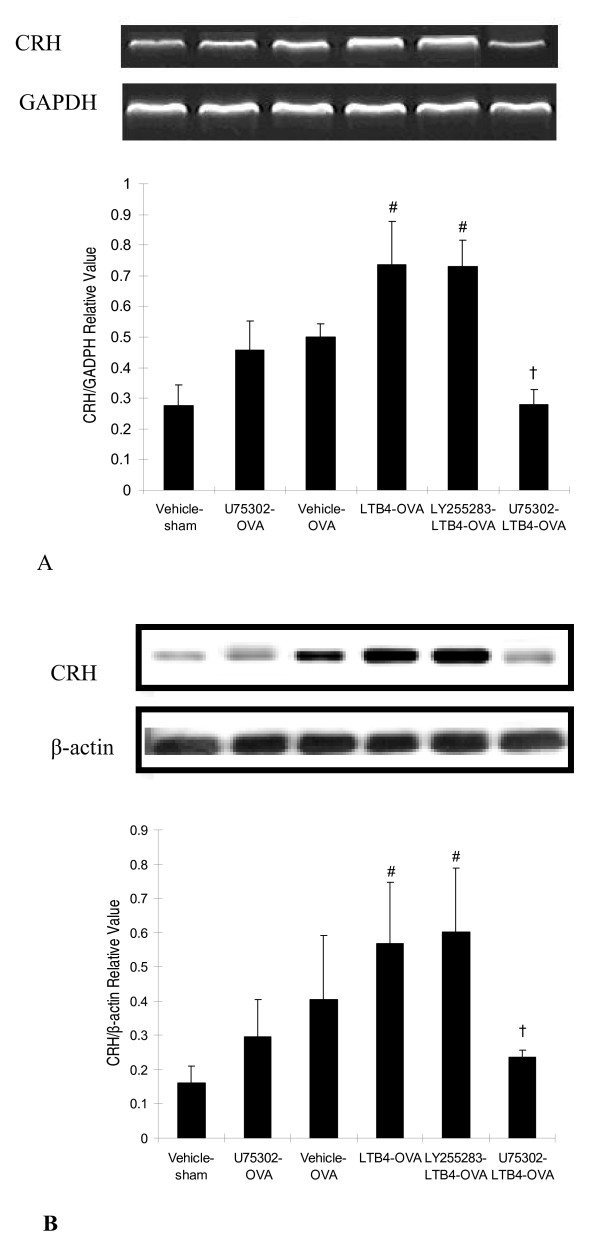
**LTB_4 _via i.c.v injection increases expression of CRH in the hypothalamus in sensitized rats**. Representative blots and densitometry analysis for expression of CRH mRNA by RT-PCR (A) and protein by Western blot (B). The blots are representative of three similar experiments, each run with independent samples. Data are expressed as the mean ± S.D. of each group (n = 3). ^#^*P *< 0.05 vs the vehicle-sham group;^†^*P *< 0.05 vs the LTB_4_-OVA or LY255283-LTB_4_-OVA group

Expression of CRH mRNA and protein in hypothalamus were evaluated in this study. We found that LTB_4 _alone via the i.c.v or OVA challenge alone markedly increased CRH mRNA and protein expression in sensitized rats. Additionally, LTB_4 _via the i.c.v further increased OVA challenge-induced CRH mRNA and protein expression in hypothalamus in sensitized rats (Fig. 5A and 5B). Furthermore, compared with LTB_4_-OVA group, U75302 alone decrease around 15% or 30% of CRH mRNA and protein expression but was not statistically significant. Pretreatment with U75302 at 10 ng via i.c.v suppressed LTB_4_-induced increase of CRH mRNA and protein expression in antigen challenged sensitized rats. On contrast, LY255283 did not significantly block the effects of LTB_4 _even at a large dose (50 ng/kg, i.c.v).

## Discussion

Recent studies have emphasized an important role of inflammatory mediators in the regulation of neuroendocrine pathways during immune challenge and in pituitary hormone secretion [17]. Particular emphasis is placed on the cross-talk between inflammation and the HPA axis. Studies have shown that inflammatory cytokines (IL-1, IL-6 etc) activate the HPA axis with the increase of secretion of cortisol, which in turn suppresses the inflammatory and immune reaction [9]. During antigen-mediated activation, CD4+ and CD8+ lymphocytes are able to produce hormones like ACTH, growth hormone (GH), thyroid stimulating hormone (TSH) and gonadotropins [18]. More directly, an antigenic challenge delivered either via the i.c.v or i.v routes evokes an increased HPA axis response in dogs that sensitized with IgE via the i.c.v route [19]. The activation of the HPA axis is also observed in allergic rhinitis during nasal provocationand and in cutaneous inflammatory disease [20,21]. Thus, the activated HPA axis inhibits the inflammation via increased cortisol. Conversely, the deregulation of the HPA axis and inability to increase glucocorticoid production in response to stress is associated with increased airway inflammation with mechanical dysfunction of the lungs in asthma. For example, in CRH-knockout mice, the airway inflammation increased and lung mechanical function decreased with the increased IL-4 and IL-13 levels, and impaired adrenal responses to stress in asthmatic mice [22]. This is also in agreement with the clinical observation that stressful life events increase the risk of a new asthma attack [23]. All these studies indicate that the crosstalk between inflammatory mediator and HPA axis regulate asthma.

Evolving evidence indicates that LTB_4 _has an important role in the development of asthma [24], and LTB_4 _mediated effects are thought through two G-protein coupled receptors (GPCRs), BLT1 (high affinity) and BLT2 (low affinity) [25,26].

Interestingly, particularly high levels of neuronal 5-LO expression and LTB_4 _content have been identified in CNS upon challenge with a variety of stimuli [27,28]. Our previous study found that antigen challenge induces the expression of 5-LO and LTA_4_H mRNA and LTB_4 _levels in brain after antigen challenge in sensitized rats. And the glucocorticoid examethasone inhibited the 5-LO and LTA4-H mRNA expression in cerebral cortex in the asthmatic rats [12]. In this study, we further evaluated the functional effect of LTB_4 _in brain through exogenously injection of LTB_4 _via i.c.v. We observed that antigen challenge or LTB_4 _injection via i.c.v alone activates the HPA axis, while combination of both further increases the HPA axis activation, which is manifested by the increased CRH mRNA and protein expression in hypothalamus, and increased ACTH and CORT level in plasma of sensitized rats. The increased HPA activation was accompanied by the alleviation of airway inflammation and improvement of lung mechanical function in sensitized rats. Using the selective receptor antagonist, we found that pretreatment of BLT1 receptor antagonist U75302 via i.c.v, but not BLT2 antagonist, completely suppressed LTB_4 _induced effects. In fact, the functional effect of LTB_4 _via i.c.v on HPA axis and peripheral asthmatic symptom in this study was supported by other studies. For example, it was found eicosaniods including LTB_4 _induces CRH secretion in explanted and cultured hypothalamus from rat [8]. And the deficiency of CRH worsen the airway inflammation and lung mechanical disfunction in sensitized mouse [22]. Base on these and aforementioned observations, we postulate that the increase of LTB_4 _level in brain induced by antigen challenge may acts as an immunomodulator, stimulates HPA axis activity via its selective BLT1 receptor, and the final increased cortisol level attenuates antigen-induced airway contraction and inflammation in the asthmatic model of rats.

It appears that different mechanisms in acute vs chronic stress as the stimulator to the HPA axis influence the inflammatory responses of the airway in asthma. It supposed that acute stress cause the activation of HPA axis and consequent cortisol release, lead to reduction of airway inflammation. However, in chronic stress condition, after continuous prolonged or intermittent stimulation, HPA axis activity is suppressed and its anti-inflammatory effect is reduced [23]. For example, studies found that acute stress or stress induced TNF-alpha or P substance production enhances airway reactivity or inflammation in OVA sensitized mice [29,30]. In adult mice, exposure to short term stress (3 days) decrease the inflammatory cell number in BALF, while the inflammatory cytokines level increased after a long-term exposure (7 days) [31]. In our study, continuous antigen attack (7 days) to sensitized rats is supposed to be the chronic stressor, but we observed the increase of CRH expression in hypothalamus, ACTH and CORT level in plasma after antigen challenge, which is contradictory to the opinion of chronic stress inhibit of HPA axis. Our postulation is that the antigen attack still provokes the acute airway response in established disease state, which may acts as acute stressor activate the NEI system and regulate the HPA axis response.

We did not find significantly differences on airway inflammation and lung mechanical function in sensitized rats treated with U75302 alone via i.c.v, which may suggest that the endogenous intracerebral LTB_4 _activity does not normally play a large role in modulating airway inflammation in this model. Notably, we observed a mild decrease (around 15%-30%) in CRH expression in hypothalamus or ACTH and CORT level in plasma after BLT1 was blocked by U75302 after antigen challenge. We postulate that the increased endogenous LTB_4 _induced by antigen challenge may mildly activate the HPA axis, but this activation of the HPA axis may be not enough to antagonize peripheral inflammation in this asthmatic model. Another possible explanation is that the functional effect of increased endogenous LTB_4 _induced by antigen challenge may be balanced by other mediators or cytokines in brain. For example, the levels of Th2- (e.g IL-4, IL-13) and Th1- cytokines (e.g INF-gamma) during asthmatic attack in brain are also changed after antigen challenge. Further studies are needed to clarify how the HPA axis response to the change of asthma-related cytokines and other inflammatory mediators, and how the HPA axis communicates with neural and endocrine networks as well as their signal pathways in regulating peripheral allergic response.

## Conclusions

This study finds that adminstration of LTB_4 _via i.c.v activates HPA axis via the BLT1 receptor, which may contribute to the attenuation of the airway inflammation and decreased lung function in asthmatic status. This study expands our concept of the regulatory role of intracranial inflammatory mediators in inflammatory diseases including asthma, and suggests a link between intracranial LTB_4 _and neuroendocrine networks. In line with this concept, these inflammatory factors probably have some favourable effects on the HPA axis of asthmatics, and may help to explain the phenomenon of self-relief after an asthmatic attack.

## Competing interests

The authors declare that they have no competing interests.

## Authors' contributions

YMD carried out the experiment design, the western blot, the RT-PCR and the manuscript writing; SJZ carried out the intracerebroventricular injection, lung function measurement, the ACTH and CORT measurement; YLZ and XWD helped to carry out the lung pathological evaluation, the ACTH and CORT measurement and performed the statistical analysis. JXJ helped to ran the RT-PCR. QMX conceived and designed this study, and helped to draft the manuscript.

## References

[B1] Frieri M Neuroimmunology and inflammation: implications for therapy of allergic and autoimmune diseases Ann Allergy Asthma Immunol 2003 90 34 40 10.1016/S1081-1206(10)61658-4 12839111

[B2] Borghetti P Saleri R Mocchegiani E Corradi A Martelli P Infection, immunity and the neuroendocrine response Vet Immunol Immunopathol 2009 130 141 62 10.1016/j.vetimm.2009.01.013 19261335 PMC7112574

[B3] Berczi I Quintanar-Stephano A Kovacs K Neuroimmune regulation in immunocompetence, acute illness, and healing Ann N Y Acad Sci 2009 1153 220 39 10.1111/j.1749-6632.2008.03975.x 19236345

[B4] Sternberg EM Neural regulation of innate immunity: a coordinated nonspecific host response to pathogens Nat Rev Immunol 2006 6 318 28 10.1038/nri1810 16557263 PMC1783839

[B5] Kelley KW Weigent DA Kooijman R Protein hormones and immunity Brain Behav Immun 2007 21 384 92 10.1016/j.bbi.2006.11.010 17198749 PMC1894894

[B6] Elenkov IJ Neurohormonal-cytokine interactions: implications for inflammation, common human diseases and well-being Neurochem Int 2008 52 40 51 10.1016/j.neuint.2007.06.037 17716784

[B7] Peters-Golden M Henderson WR Jr Leukotrienes N Engl J Med 2007 357 1841 54 10.1056/NEJMra071371 17978293

[B8] Bernardini R Chiarenza A Calogero AE Gold PW Chrousos GP Arachidonic acid metabolites modulate rat hypothalamic corticotropin-releasing hormone secretion in vitro Neuroendocrinology 1989 50 708 15 10.1159/000125303 2559344

[B9] Chrousos GP The hypothalamic pituitary adrenal axis and immunemediated inflammation N Engl J Med 1995 332 1351 1362 10.1056/NEJM199505183322008 7715646

[B10] Xie QM Chen JQ Shen WH Yang QH Bian RL Comparison of bronchodilating and antiinflammatory activities of oral formoterol and its (R,R)-enantiomers Acta Pharmacol Sin 2003 24 277 82 12617779

[B11] Deng YM Xie QM Chen JQ Bian RL Coincidental increase of leukotriene B4 between cerebral cortex and lung tissue of sensitized rats Acta Pharmacol Sin 2003 24 1039 44 14531949

[B12] Deng YM Xie QM Zhang SJ Chen JQ Yang QH Bian RL Changes of 5-lipoxygenase pathway and proinflammatory mediators in cerebral cortex and lung tissue of sensitized rats Acta Pharmacol Sin 2005 26 353 8 10.1111/j.1745-7254.2005.00043.x 15715933

[B13] Mauser PJ Edelman NH Chapman RW Central nervous system control of airway tone in guinea pigs: the role of histamine J Appl Physiol 1988 65 2024 9 2905361 10.1152/jappl.1988.65.5.2024

[B14] Xie QM Wu X Wu HM Deng YM Zhang SJ Zhu JP Oral administration of allergen extracts from Dermatophagoides farinae desensitizes specific allergen-induced inflammation and airway hyperresponsiveness in rats Int Immunopharmacol 2008 8 1639 45 10.1016/j.intimp.2008.07.015 18721904

[B15] Duan W Chan JH Wong CH Leung BP Wong WS Anti-inflammatory effects of mitogen-activated protein kinase kinase inhibitor U0126 in an asthma mouse model J Immunol 2004 172 7053 9 15153527 10.4049/jimmunol.172.11.7053

[B16] Meloni EG Jackson AV Cohen BM Carlezon WA Jr Corticotropin-releasing factor from the rat brain measured by protein immunoblot Peptides 2005 26 2252 2256 10.1016/j.peptides.2005.04.011 15978700

[B17] Callewaere C Banisadr G Rostene W Parsadaniantz SM Chemokines and chemokine receptors in the brain: implication in neuroendocrine regulation J Mol Endocrinol 2007 38 355 63 10.1677/JME-06-0035 17339398

[B18] Mocchegiani E Santarelli L Costarelli L Cipriano C Muti E Giacconi R Malavolta M Plasticity of neuroendocrine-thymus interactions during ontogeny and ageing: role of zinc and arginine Ageing Res Rev 2006 5 281 309 10.1016/j.arr.2006.06.001 16904953

[B19] Matsumoto I Inoue Y Shimada T Aikawa T Brain mast cells act as an immune gate to the hypothalamic-pituitary-adrenal axis in dogs J Exp Med 2001 194 71 8 10.1084/jem.194.1.71 11435473 PMC2193441

[B20] Kalogeromitros D Syrigou EK Makris M Kempuraj D Stavrianeas NG Vasiadi M Theoharides TC Nasal provocation of patients with allergic rhinitis and the hypothalamic-pituitary-adrenal axis Ann Allergy Asthma Immunol 2007 98 269 73 10.1016/S1081-1206(10)60717-X 17378259

[B21] O'Kane M Murphy EP Kirby B The role of corticotropin-releasing hormone in immune-mediated cutaneous inflammatory disease Exp Dermatol 2006 15 143 53 10.1111/j.1600-0625.2006.00382.x 16480421

[B22] Silverman ES Breault DT Vallone J Subramanian S Yilmaz AD Mathew S Subramaniam V Tantisira K Pacak K Weiss ST Majzoub JA Corticotropin-releasing hormone deficiency increases allergen-induced airway inflammation in a mouse model of asthma J Allergy Clin Immunol 2004 114 747 754 10.1016/j.jaci.2004.06.055 15480311

[B23] Priftis KN Papadimitriou A Nicolaidou P Chrousos GP Dysregulation of the stress response in asthmatic children Allergy 2009 64 18 31 10.1111/j.1398-9995.2008.01948.x 19132973

[B24] Hallstrand TS Henderson WR Jr An update on the role of leukotrienes in asthma Curr Opin Allergy Clin Immunol 2010 10 60 6 10.1097/ACI.0b013e32833489c3 19915456 PMC2838730

[B25] Yokomizo T Izumi T Chang K Takuwa Y Shimizu T A G-protein-coupled receptor for leukotriene B4 that mediates chemotaxis Nature 1997 387 620 624 10.1038/42506 9177352

[B26] Yokomizo T Kato K Terawaki K Izumi T Shimizu T A new therapeutic target in inflammation and immunological disorders J Exp Med 2000 192 421 432 10.1084/jem.192.3.421 10934230 PMC2193217

[B27] Lammers CH Schweitzer P Facchinetti P Arrang JM Madamba SG Siggins GR Piomelli D Arachidonate 5-lipoxygenase and its activating protein: prominent hippocampal expression and role in somatostatin signaling J Neurochem 1996 66 147 52 8522947 10.1046/j.1471-4159.1996.66010147.x

[B28] Matsuo M Hamasaki Y Fujiyama F Miyazaki S Eicosanoids are produced by microglia, not by astrocytes, in rat glial cell cultures Brain Res 1995 685 201 4 10.1016/0006-8993(95)00490-H 7583247

[B29] Joachim RA Quarcoo D Arck PC Herz U Renz H Klapp BF Stress enhances airway reactivity and airway inflammation in an animal model of allergic bronchial asthma Psychosom 2003 65 811 815 10.1097/01.PSY.0000088582.50468.A3 14508025

[B30] Joachim RA Sagach V Quarcoo D Dinh T Arck PC Klapp BF Upregulation of tumor necrosis factor-alpha by stress and substance p in a murine model of allergic airway inflammation Neuroimmunomodulation 2006 13 43 50 10.1159/000094394 16837794

[B31] Forsythe P Ebeling C Gordon JR Befus AD Vliagoftis H Opposing effects of short- and long-term stress on airway inflammation Am J Respir Crit Care Med 2004 169 220 226 10.1164/rccm.200307-979OC 14604839

